# New dammarane-type triterpenoids from hydrolyzate of total *Gynostemma pentaphyllum* saponins with protein tyrosine phosphatase 1B inhibitory activity

**DOI:** 10.1080/14756366.2023.2281263

**Published:** 2023-11-15

**Authors:** Daopeng Tan, Jianmei Wang, Xianting Wang, Lin Qin, Yimei Du, Changkuo Zhao, Peijun Liu, Qianru Zhang, Feifei Ma, Jian Xie, Di Wu, Yuqi He

**Affiliations:** a Guizhou Engineering Research Center of Industrial Key-technology for Dendrobium Nobile, Zunyi Medical University, Zunyi, Guizhou, China; b Joint International Research Laboratory of Ethnomedicine of Ministry of Education, Zunyi Medical University, Zunyi, Guizhou, China

**Keywords:** *Gynostemma pentaphyllum*, dammarane triterpenoids, protein tyrosine phosphatase 1B, phytochemistry

## Abstract

Protein tyrosine phosphatase 1B (PTP1B) is a key factor and regulator of glucose, lipid metabolism throughout the body, and a promising target for treatment of type 2 diabetes mellitus (T2DM). *Gynostemma pentaphyllum* is a famous oriental traditional medicinal herbal plant and functional food, which has shown many beneficial effects on glucose and lipid metabolism. The aim of the present study is to assess the inhibitory activity of five new and four known dammarane triterpenoids isolated from the hydrolysate product of total *G. pentaphyllum* saponins. The bioassay data showed that all the compounds exhibited significant inhibitory activity against PTP1B. The structure-activity relationship showed that the strength of PTP1B inhibitory activity was mainly related to the electron-donating group on its side chain. Molecular docking analysis suggested that its mechanism may be due to the formation of competitive hydrogen bonding between the electron-donating moiety and the Asp48 amino acid residues on the PTP1B protein.

## Introduction

Diabetes mellitus is a chronic metabolic disease caused by insufficient insulin secretion or insulin resistance, with hyperglycaemia as the typical clinical symptom, which can lead to a series of complications such as retinopathy and cardiovascular disease, and even life-threatening in severe cases^1^. Type 2 diabetes mellitus accounts for the majority of diabetic patients, mainly due to insulin resistance, which reduces the sensitivity of target cells to insulin and the uptake of glucose capacity, which ultimately leads to elevated glucose levels in the blood^1^. Although hypoglycaemic agents have been widely used, these agents have a great many limitations, including adverse effects and high rates of secondary failure^2^. With the continuous research on the physiological function of insulin, a number of new targets for the treatment of diabetes have been discovered. Among them, protein-tyrosine phosphatase 1B (PTP1B) is an important negative regulator of insulin signalling and plays an important role in the process of insulin signalling^3^. The sensitivity of target cells to insulin can be improved by inhibiting the activity of PTP1B^4^. Therefore, it has been suggested that inhibition of PTP1B is an effective therapeutic approach to treatment of type-2 diabetes mellitus and obesity^5–8^.

*Gynostemma pentaphyllum* (Thunb.) Makino (Cucurbitaceae), usually called “Jiaogulan” in China, is also widely distributed in Asia including Japan, Korea and other southeast Asian countries^9–11^. In China, *G. pentaphyllum* has been prescribed in Chinese medicines to treat various diseases, especially diabetes, referred as “thirsty disease” for a long history from the Ming Dynasty (1368–1644 AD)^12^^,^^13^ and mainly cultivated and became an important income resource for local farmers in Shaanxi, Guangxi, Fujian, Guizhou and Hubei province, today^14^^,^^15^. Previous phytochemical investigations indicated that the dammarane triterpenoid and flavonoid glycosides were the major constituents in *G. pentaphyllum*^16^^,^^17^. To date, more than 180 gypenosides, along with flavonoids were isolated from the plant exhibiting various biological activities such as antioxidant, anti-inflammatory, antitumor, antidiabetic and hepatoprotective effects^18–22^. The structure-activity relationship studies indicated that as the presence of sugar moieties in the saponins, aglycones were more effective than glycosides^23^. Previously, it was reported in the literature that some triterpenoids isolated from the hydrolysis products of *G. pentaphyllum* total saponins were found to have inhibitory activity against PTP1B^24^^,^^25^.

In the present study, to further search for bioactive aglycones, a hydrolyzate of *G. pentaphyllum* total saponins was investigated, and led to the isolation of five new triterpene derivative **1**–**5**, together with four known analogues **6**–**9**. Their structures were elucidated using spectroscopic methods including HR-ESI-MS, 1D and 2D NMR or X-ray crystallography. Among them, compound **1** was an unpredictable new skeleton dammarane triterpenoids. In addition, the inhibitory activity of these triterpenoids against PTP1B were evaluated.

## Materials and methods

### General experimental procedures

In the separation process, the column chromatography as performed by silica-gel (100–200 and 300–400 mesh, Qingdao Marine Chemical Co., Qingdao, China), and Sephadex LH-20 (GE Healthcare, Marlborough, MA). The TLC detection was carried by heating a silica gel plate (Qingdao Marine Chemical Co., Qingdao, China) spayed with 10% H_2_SO_4_ in ethanol. The 1D and 2D NMR spectra were recorded on a Varian INOVA AS 400 instrument (Agilent, Santa Clara, USA) with TMS as the internal standard. The chemical shift was shown in ppm while coupling constant *J* in Hz. Deuterated solvent (CD_3_Cl) was purchased from Sigma Aldrich for NMR analysis. HR-ESI-MS data were collected by a UPLC–Q/TOF-MS system including a 1290 Infinity II UPLC system (Agilent, USA) and an Agilent 6545 Q/TOF-MS system (Agilent, MA, USA). Optical rotations were measured by a JASCO P-1020 polarimeter (Kyoto, Japan). The single crystal X-ray diffraction data were obtained by a SuperNova, Dual, Cu at zero, AtlasS2 diffractometer (Rigaku, Japan). Besides, other analytical grade chemical solvent was obtained from Jinhuada Chemical Co., ltd. Guangzhou, P.R. China. Recombinant human PTP1B protein (ab51277) was purchased from Abcam (Shanghai) Trading Co., Ltd. All chemical reagents used in the enzymatic assays were produced by Laibo Chemicals Industries, Ltd.

### Plant material

The total saponins extract (>80%, by UV) of *G. pentaphyllum* (Thunb.) Makino was purchased from Shaanxi Zhongxin biotech Co. Ltd., in 2019 and authenticated by Associate Professor Daopeng Tan (Pharmacognosy, Zunyi Medical University). A voucher specimen (No. 20190501) was deposited in the Herbarium of Zunyi Medical University.

### Acid hydrolysis and isolation

The crude of saponins (500 g) of *G. pentaphyllum* was dissolved by MeOH (1000 ml) and then hydrolysed by adding 10% HCl (500 ml) at 50 °C for 8 h^13^. After the evaporation under reduced pressure and filtration, the crude gypenoside aglycones residue (150 g) was obtained. The residue was subjected to silica gel column chromatography and eluted with petroleum ether/EtOAC (50:1–1:1) and CH_2_Cl_2_/MeOH (50:1–1:1) gradient to afford ten fractions (Fr. A-J). Fr. B (2.5 g) was fractionated by silica gel CC with n-hexane/EtOAC (50:1) to yield compound **1** (15 mg), **2** (23 mg), **6** (60 mg) and **7** (55 mg). Fr. C (1.8 g) was purified over silica gel CC with petroleum ether/acetone (40:1) to afford compound **3** (12 mg), **8** (45 mg) and **9** (50 mg). Fr. D (2.3 g) was separated by silica gel CC with n-hexane/acetone (30:1) to obtain compound **4** (11 mg) and **5** (17 mg).

Gypensapogenin I (**1**): White amorphous powder; [α]${}_{D}^{25}$= −9.8° (c 0.08, MeOH); The ^1^H NMR (400 MHz, CD_3_Cl) and ^13^C NMR (100 MHz, CD_3_Cl) spectral data was shown in Table 1; HR-ESI-MS: m/z 453.3368 [M + H]^+^ (calcd. 453.3368, for C_30_H_45_O_3_).

**Table 1.** ^1^H and ^13^C NMR data of compounds 1–5 (in CD_3_Cl, J in Hz at 400 MHz and 100 MHz).

No.	1		2		3		4		5	
	*δ_H_*	*δ_C_*	*δ_H_*	*δ_C_*	*δ_H_*	*δ_C_*	*δ_H_*	*δ_C_*	*δ_H_*	*δ_C_*
1	4.38, brs	73.8	0.95,1.65, m	39.0	0.90,1.55,m	40.8	0.92,1.60,m	40.8	0.95,1.65, m	39.1
2	1.78, 1.90, m	24.0	1.23,1.61, m	27.3	1.55,2.10	27.4	1.27,2.02,m	26.8	0.92,1.85,m	27.7
3	3.82, m	83.9	3.17	78.8	3.43	79.2	3.43	79.2	3.17	78.9
4		39.1		40.4		40.7		40.7		40.3
5		130.0	0.71	55.7	0.82	57.5	0.82	57.5	0.68,m	55.8
6	1.94, m	20.2	1.31,1.51, m	18.2	1.42,1.54,m	20.0	1.35,1.55,m	19.9	1.21,1.48, m	18.2
7	1.78, m	32.0	1.27,1.55, m	35.4	1.25,1.55,m	37.0	1.23,1.55,m	36.9	1.21,1.49, m	35.2
8		38.3		38.9		41.9		41.9		38.9
9	2.20, m	37.5	1.26,m	50.7	1.35,m	52.3	1.35,m	52.5	1.55,m	43.9
10		132.8		37.1		38.6		38.6		37.5
11	1.10 1.71, m	25.9	1.50,m	21.2	1.43,m	22.9	1.50,m	22.8	1.45,m	21.2
12	1.60, 1.90, m	25.3	1.61,2.00, m	28.5	1.15,1.70,m	32.3	1.23,1.85,m	29.9	1.69,1.43, m	25.1
13	2.10, m	44.9	1.71,m	46.9	2.22,m	42.4	2.03	45.4	2.98,m	41.6
14		48.3		50.0		51.8		51.0		49.5
15	1.2, 1.6, m	25.2	1.17,1.65, m	31.5	0.97,1.81,m	29.9	1.05,1.50,m	32.5	1.03,1.43,m	30.8
16	1.2, 1.7, m	26.4	1.15,1.55, m	25.0	1.65,2.15,m	25.7	1.6,2.01,m	26.9	1.69,1.43, m	25.1
17	3.15, m	47.1	2.55,m	44.1	2.50,m	50.09	2.15,m	44.0	1.83,m	42.1
18	0.83, s	22.5	0.82,s	16.2	0.93,s	17.0	0.97,s	17.0	0.89,s	15.8
19	1.8, m	32.6	0.95,s	15.5	0.79,s	17.7	0.84,s	17.7	0.81,s	16.2
20		201.0		175.6		79.2	3.56,m	43.7	1.25,m	50.8
21		149.9	3.04,s	35.2		181.0		178.6		179.8
22	5.88, t (5.0)	106.8	6.02,s	132.0	2.15,m	41.1	2.85,3.56,	47.1	2.91, 2.40,dd (20.0, 4.0)	44.0
23	2.15, m	19.0		197.4	2.3,2.65,m	24.6		211.4		198.7
24	1.25, 1.55, m	31.9		129.4	5.3,t,	126.4	2.94,s	57.2	6.05,s	123.4
25		74.3		144.6		132.6		70.7		155.9
26	1.25, s	31.1	1.85,s	24.0	1.59,s	18.8	1.51,s	31.2	1.85,s	27.3
27	1.23 s	26.5	2.26,s	19.6	1.65,s	27.0	1.50,s	31.7	2.1,s	20.8
28	1.12 s	27.1	0.95,s	27.9	1.23,s	29.5	1.23,s	29.5	0.93,s	27.9
29	0.84, s	15.0	0.75,s	15.3	1.03,s	17.6	1.03,s	17.6	0.74,s	15.5
30	0.86, s	12.6	0.89,s	15.8	0.98,s	17.8	0.88,s	17.3	0.80,s	15.4

Gypensapogenin II (**2**): White amorphous powder; [α]${}_{D}^{25}$= −38.4° (c 0.1, MeOH); The ^1^H NMR (400 MHz, CD_3_Cl) and ^13^C NMR (100 MHz, CD_3_Cl) spectral data was shown in Table 1; HR-ESI-MS: m/z 439.3577 [M + H]^+^ (calcd. 439.3576, for C_30_H_47_O_2_).

Gypensapogenin III (**3**): White amorphous powder; [α]${}_{D}^{25}$= −24.8° (c 0.1, MeOH); The ^1^H NMR (400 MHz, CD_3_Cl) and ^13^C NMR (100 MHz, CD_3_Cl) spectral data was shown in Table 1; HR-ESI-MS: m/z 492.4057 [M + NH_4_]^+^ (calcd. 492.4053, for C_30_H_54_O_4_N).

Gypensapogenin IV (**4**): White amorphous powder; [α]${}_{D}^{25}$= −37.4° (c 0.1, MeOH); The ^1^H NMR (400 MHz, CD_3_Cl) and ^13^C NMR (100 MHz, CD_3_Cl) spectral data was shown in Table 1; HR-ESI-MS: m/z 473.3635 [M-H_2_O + H]^+^ (calcd. 473.3631, for C_30_H_49_O_4_).

Gypensapogenin V (**5**): White amorphous powder; [α]${}_{D}^{25}$= −19.4° (c 0.1, MeOH); The ^1^H NMR (400 MHz, CD_3_Cl) and ^13^C NMR (100 MHz, CD_3_Cl) spectral data was shown in Table 1; HR-ESI-MS: m/z 473.3650 [M + H]^+^ (calcd. 473.3631, for C_30_H_49_O_4_).

### Protein tyrosine phosphatase 1B inhibition assay

The enzymatic assays of protein tyrosine phosphatase 1B (PTP1B) were referred ^26^. The PTP1B inhibitory activities of test compounds were examined in 96-well plates using the colorimetric method. Each test compounds and NaVO_4_ (positive control) were dissolved separately in DMSO and then diluted into gradient concentrations with MOPS solution (25 mM MOPS, 2 mM dl-dithiothreitol (DTT), 1 mM EDTA, 0.1 M NaCl, pH = 7.0). The reaction system was 100 μL: MOPS (25 mM, pH = 7.0), DTT (2 mM), EDTA (1 mM, pH = 7.0), NaCl (0.1 M), PTP 1B (25 nM), and disodium-4-nitrophenyl phosphate (pNPP, 2 mM), and the enzyme-catalysed reaction was terminated by the addition of 1.0 M NaOH after incubation at 37 °C for 30 min. The inhibition efficiency of the compounds on the enzymatic activity of PTP1B was assayed by detecting the amount of the hydrolysed product, p-nitrophenol (p-NP) at 405 nm using a microplate reader. The PTP1B inhibition activity was expressed as inhibition (%) and was calculated as Equation (1):

(1) $$\text{Inhibition }\left(\%\right)=(1-\Delta A_{\text{sample}/}\Delta A_{\text{control}})\times 100\%$$

### Protein tyrosine phosphatase 1B inhibition kinetic assay

The kinetic behaviour of the active compounds against PTP1B and the corresponding inhibition constants (*K*i values) were investigated using Lineweaver-Burk double reciprocal plots and Dixon plots. Different concentrations of p-NPP substrates (1, 2, 5 and 10 mM) were subjected to enzymatic reactions with various concentrations of active compounds (0, 0.5, 1.0 and 5.0 μM for 18) and 100 nM PTP1B in 96-well plates. The absorbance of the reaction mixture was recorded every 3 min by a plate reader. The enzymatic velocity of the enzyme reaction was calculated based on the time-Δabs plot. All assays were performed in triplicate.

### Molecular docking study

Dammarane triterpenoids with optimal PTP1B inhibitory activity was selected for molecular docking with PTP1B protein. The PTP1B protein crystal structure was downloaded from the AlphaFold Protein Structure Database (https://alphafold.ebi.ac.uk) and modified by the Autodock tools 1.5.6 software^27^^,^^28^. The PTP1B protein molecule was optimised for dehydrogenation, hydrogenation, etc., and defined its active site including Tyr46, Asp48, Phe182, Cys215, Ser216, Ile219, Arg221, Met258, Gln262, Gln266^29^. The 3D structure of preferred dammarane triterpenoids were built by ChemBioDraw Ultra14.0 and then converted to PDBQT coordinateds using AutoDockTools. The rotatable bonds of the ligand were assigned through AutoDock Tools, and the ligand docking was performed by the AutoDock Vina. Construct 2D maps of protein-ligand interactions using LigPlot software to analyse the interaction forces between protein and ligand binding. Construct 3D maps of protein-ligand interactions using PyMOL software to show the binding sites between proteins and ligands.

### ADME analysis

SwissADME which is a web-based tool was applied for ADME analysis. SwissADME helped us to predict the ADME parameters, pharmacokinetic qualities, drug-like properties and medicinal chemistry friendliness of dammarane triterpenoids of *G. pentaphyllum* by utilising its ability to compute physicochemical descriptors^30^.

## Results and discussion

### Structure elucidation

Five unreported dammarane triterpenoids **1–5** together with four known analogues **6–9** were isolated from the hydrolyzate of total *G. pentaphyllum* saponins. Structures of undescribed triterpenoids **1–5** were elucidated on the basis of spectral data or single crystal X-ray diffraction data interpretation, whereas the structures of four known compounds **6–9** were confirmed by comparing their spectral data in literature^23^.

**Figure 1.**
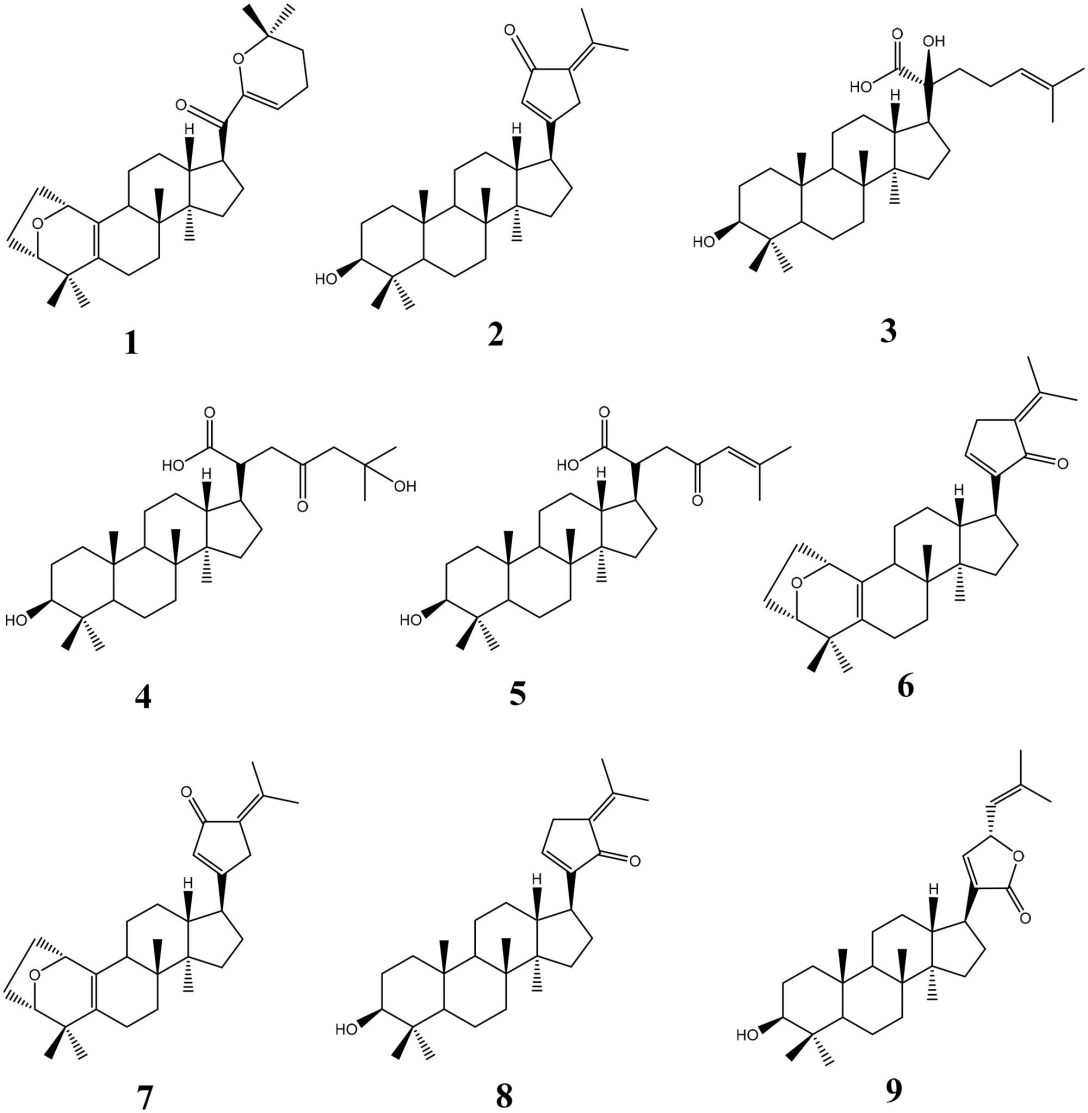
Chemical structures of compounds **1**–**9**.

**Figure 2.**
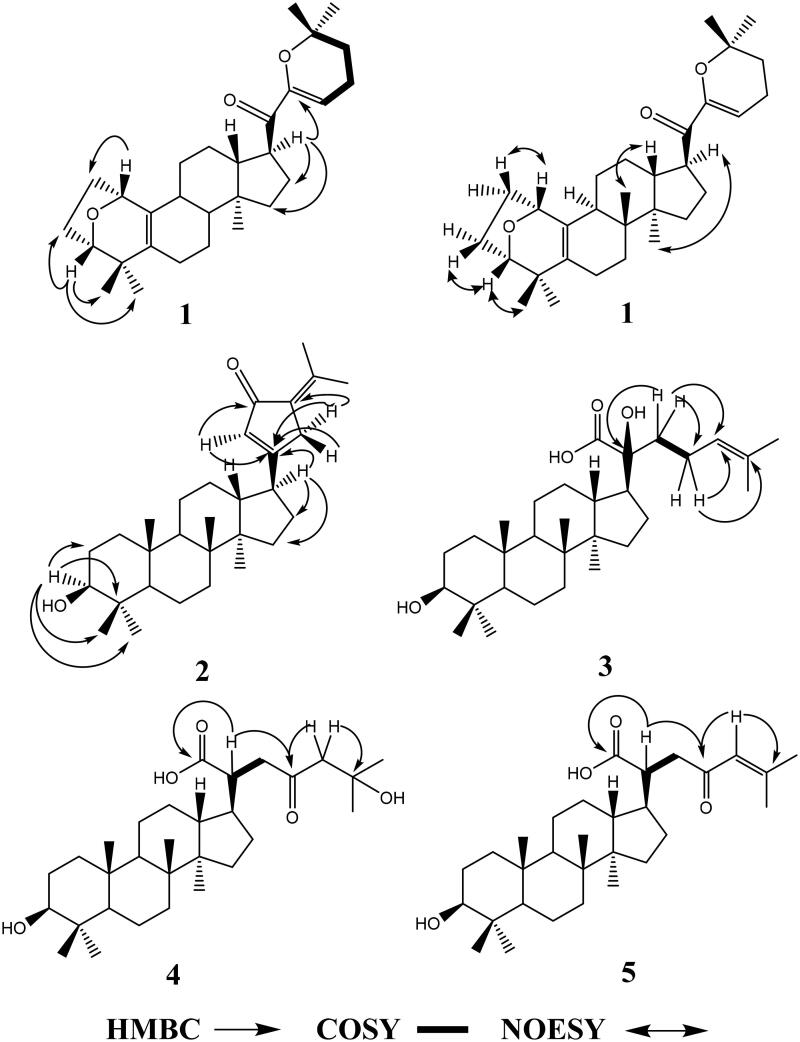
HMBC, COSY and NOESY correlations of compounds **1**–**5**.

**Figure 3.**
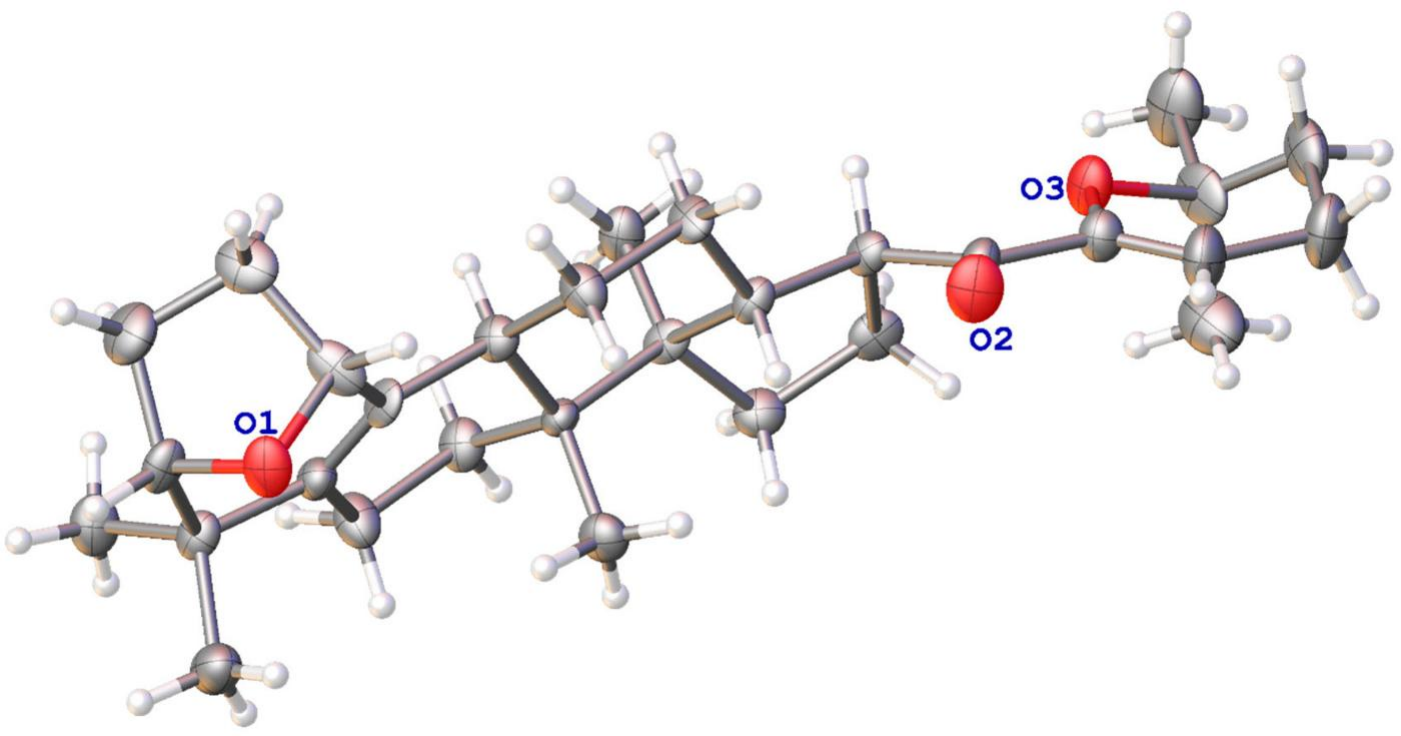
Crystal structure of compounds **1**.

**Scheme 1.**
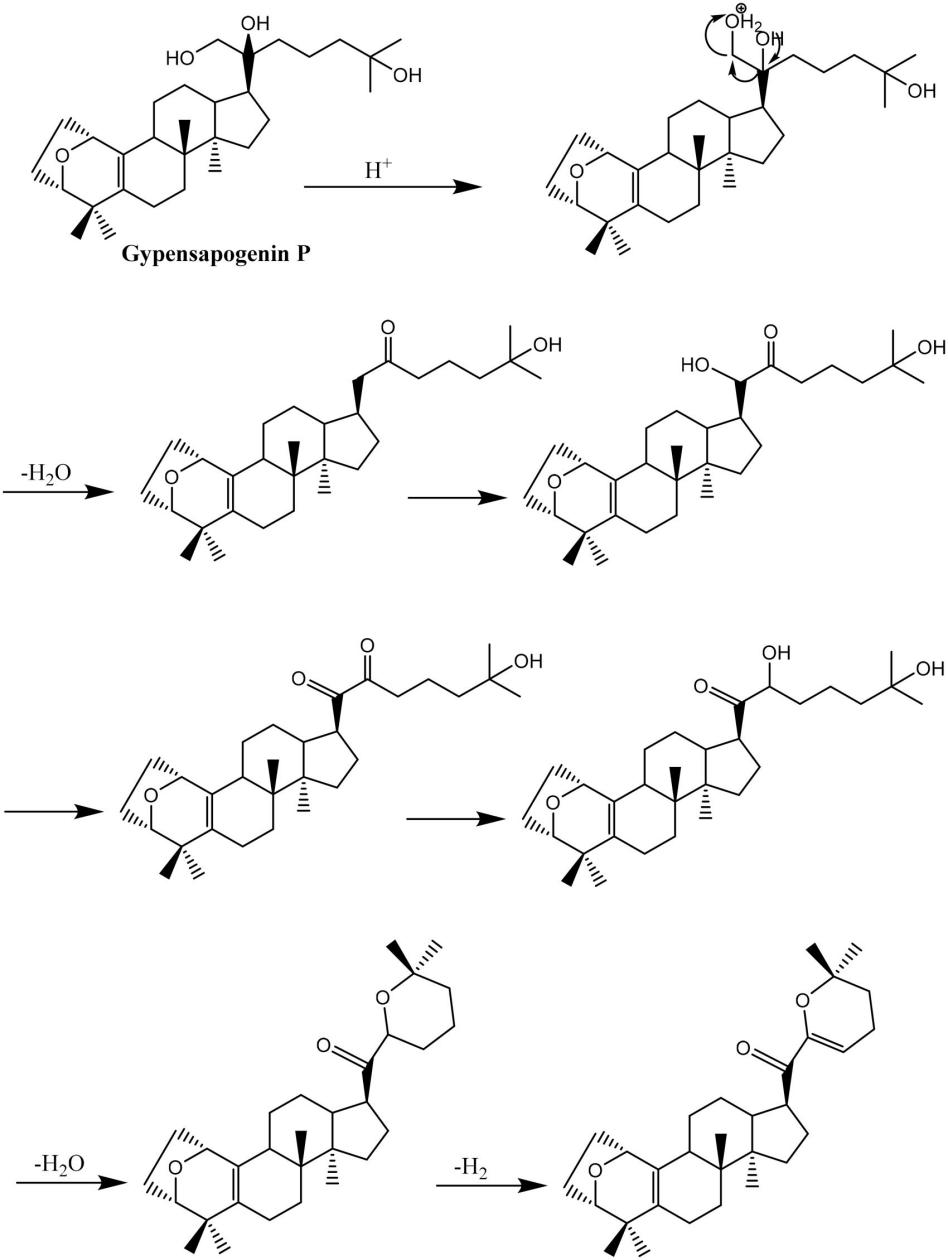
Hypothetical mechanism for the formation of ring F in compound **1**.

Gypensapogenin I (**1**) was isolated as a white amorphous powder with a molecular formula of C_30_H_44_O_3_ as determined by the HR-ESI-MS at m/z 453.3368 [M + H]^+^, (calcd. for 453.3368) and NMR data, requiring ten degrees of unsaturation (Table 1). The ^1^H NMR spectrum showed signals due to an olefinic proton (*δ*: 5.87), two oxygen-bearing protons (*δ*: 4.38, 3.83) and six methyl groups (*δ*: 1.25, 1.23, 1.12, 0.86, 0.83, 0.82). The ^13^C NMR spectrum showed 30 carbon resonances and demonstrated the presence of an unsaturated ketone by the signals at *δ*: 201.0, 149.9, 132.8, 130.0, 106.8. So, compound **1** was determined as a sapogenin from the hydrolized saponins of *G. pentaphyllum*. Furthermore, the NMR spectrum of compound **1** closely resembled that of Gypensapogenin A and B in the high field^23^. Those suggested that the structure of compound **1** was similar to Gypensapogenin A and B with a 7-membered cycle, an oxygen bridge and a double bond. In the HMBC spectrum, the long-range correlations from *δ*: 5.88 (H-22) to *δ*: 201.0 (C-20), 149.4 (C-21), 19.0 (C-23) and 31.9 (C-24), *δ*:2.15 (H-23) to *δ*: 149.4 (C-21), 106.8 (C-22), 31.9 (C-24) and 74.3 (C-25), *δ*:1.25, 1.23 (H-26, 27) to *δ*: 74.3 (C-25), 31.9 (C-24), respectively, indicated the existence of a dihyro-pyran cycle with two methyl groups in the side chain. With the help of HSQC, COSY and NOESY data, compound **1** was elucidated as Figures 1 and 2 and named Gypensapogenin I. The chemical structure was finally confirmed by the single crystal X-ray diffraction analysis (flack parameter −0.7 (5), Figure 3). Additionally, the structure of compound **1** was demonstrated as an unprecedented dammarane triterpene skeleton characterised as C-21 was inserted into the side chain. The possible formation of Gypensapogenin I was deduced as shown in Scheme 1.

**Crystal Data** for Gypensapogenin I, C_30_H_44_O_3_ (*M* = 452.65 g/mol), orthorhombic, space group P2_1_2_1_2_1_ (no. 19), *a* = 6.1257(6) Å, *b* = 9.1748(10) Å, *c* = 45.065(5) Å, *V* = 2532.7(5) Å^3^, *Z* = 4, *T* = 150.00(10) K, μ(Cu Kα) = 0.575 mm^−1^, *Dcalc* = 1.187 g/cm^3^, 7830 reflections measured (3.922° ≤ 2Θ ≤ 148.812°), 4370 unique (*R*_int_ = 0.0546, R_sigma_ = 0.0659) which were used in all calculations. The final *R*_1_ was 0.1277 (I > 2σ(I)) and *wR*_2_ was 0.3306 (all data).

Gypensapogenin II (**2**) was isolated as a white amorphous powder with a molecular formula of C_30_H_46_O_2_ as determined by the HR-ESI-MS at m/z 439.3577 [M + H]^+^, (calcd. for 439.3576) and NMR data, requiring eight degrees of unsaturation (Table 1). The ^1^H NMR spectrum showed signals due to an olefinic proton (*δ*: 6.02), one oxygen-bearing protons (*δ*: 3.17) and seven methyl groups (*δ*: 2.26, 1.85, 0.95, 0.95, 0.89, 0.82, 0.75). The ^13^C NMR spectrum showed 30 carbon resonances and demonstrated the presence of a ketone carbonyl at the signals of *δ*: 197.4 and a pair of alkenyl carbon signals at *δ*: 129.4 and 144.6. So, compound **2** was elucidated to be a triterpene, which was very similar with Gypensapogenin C in the ^1^H and ^13^C NMR spectrum data^23^. Analysis of HMBC, HMQC, COSY and NOESY spectrum led to the elucidation of the structure of compound **2** named Gypensapogenin II (Figures 1 and 2).

Gypensapogenin III (**3**) was isolated as a white amorphous powder with a molecular formula of C_30_H_50_O_4_ as determined by the HR-ESI-MS at m/z 492.4057 [M + NH_4_]^+^, (calcd for 492.4053) and NMR data, requiring six degrees of unsaturation (Table 1). In the ^1^H NMR spectrum, seven methyl group signals at *δ*:1.65, 1.59, 1.23, 1.03, 0.98, 0.93, 0.79, one olefinic proton at *δ*:5.30, and one oxygen bearing proton at *δ*:3.43 were observed. And in the ^13^C NMR spectrum, 30 carbon resonances signals were observed. Among them, one ester carbonyl, a group of allylic and two oxygen bearing carbons were presented at the signals of *δ*: 181.0, 132.6, 126.4, 79.2 × 2, respectively. On the basis of literature^25^^,^^31^, the ^1^H and ^13^C NMR spectrum data of compound **3** were very similar to that of (3*β*,20*S*,23*R*)-3,20,23-trihydroxydammar-24-en-21-oic acid-21,23-lactone except for the changes in the signals of H-24 and C-23. Interpretation of HMBC, HMQC, COSY and NOESY spectrum, the structure of compound **3** was identified as Figures 1 and 2 and named Gypensapogenin III.

Gypensapogenin IV (**4**) was isolated as a white amorphous powder with a molecular formula of C_30_H_50_O_5_ as determined by the HRESIMS at m/z 473.3635 [M-H_2_O + H]^+^, (calcd for 473.3631) and NMR data, requiring six degrees of unsaturation (Table 1). Compound **4** exhibited NMR spectrum features similar to those of compound **3** except for the inexistence of allylic carbons replaced by more one ketone carbonyl in the ^1^H and ^13^C NMR spectrum. On the basis of HMBC, HMQC, COSY and NOESY spectrum, the structure of compound **4** was elucidated as Figures 1 and 2 and named Gypensapogenin IV.

Gypensapogenin V (**5**) was isolated as a white amorphous powder with a molecular formula of C_30_H_48_O_4_ as determined by the HRESIMS at m/z 473.3650 [M + H]^+^, (calcd for 473.3631) and NMR data, requiring seven degrees of unsaturation (Table 1). Compound **5** exhibited the NMR spectrum features similar to those of compound **3** except that there was one less oxycarbon atom and one more carbonyl carbon atom in the ^1^H and ^13^C NMR spectrum. On the basis of HMBC, HMQC, COSY and NOESY spectrum, the structure of compound **5** was elucidated as Figures 1 and 2 and named Gypensapogenin V.

### Biological evaluation

The pNPP method was used to examine their PTP1B inhibitory activities of these nine dammarane derivatives isolated from *G. pentaphyllum*. The results showed that all these nine compounds exhibited some PTP1B inhibitory activity and compounds **2**, **3**, **4**, **9** showed more inhibitory activity against PTP1B than the positive control (NaVO_4_) (Table 2).

Comparing the structures of these compounds with their inhibitory activities against PTP1B, we can find such conformational relationships. firstly, the presence of -OH in C-3 is favourable for the inhibitory activity of PTP1B, however, in compounds **1**, **6**, **7**, the spatial site-blocking formed by the seven-membered ring A reduces their activities. Secondly, in the side chain, the presence of electron-donating (–OH, –COOH) is favourable for their inhibitory activity, whereas the presence of electron-withdrawing groups such as carbonyl group, double bond, etc. is unfavourable for its inhibitory activity.

### Kinetic characterisation

**Figure 4.**
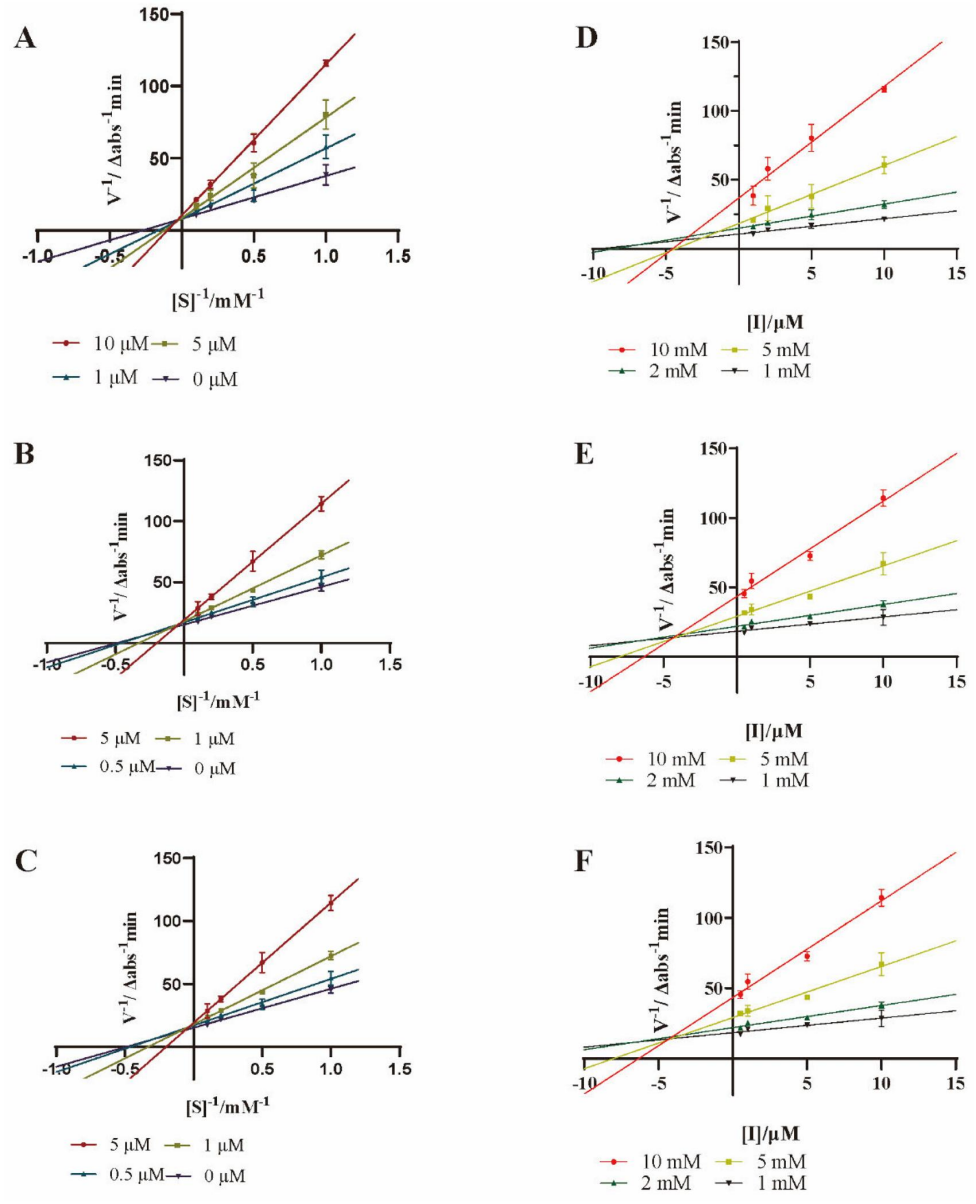
Lineweaver-Burk plots for PTP1B inhibition of compounds **3** (A), **4** (B) and **9** (C). Dixon plots for PTP1B inhibition of compounds **3** (D), **4** (E) and **9** (F). Each value was expressed as means ± SD of three replications.

Lineweaver − Burk double reciprocal plot and Dixon plot were further applied to study the kinetic behaviour of compounds **3**, **4** and **9** against PTP1B. As shown in Figure 4(A–C), the maximum rate of the PTP1B catalysed reaction was not changed, and the *K*_m_ was increased with the increase of concentrations. Therefore, compounds **3**, **4** and **9** are competitive inhibitors against PTP1B. In the Dixon plot method (Figure 4(D–F)), the *K*_i_ values of **3**, **4** and **9** were determined to be 1.16 ± 0.47 μM, 2.44 ± 0.09 μM and 5.58 ± 0.70 μM, respectively. The relatively lower *K*_i_ value of **3** makes it to be a promising competitive PTP1B inhibitor.

### Molecular docking analysis

**Figure 5.**
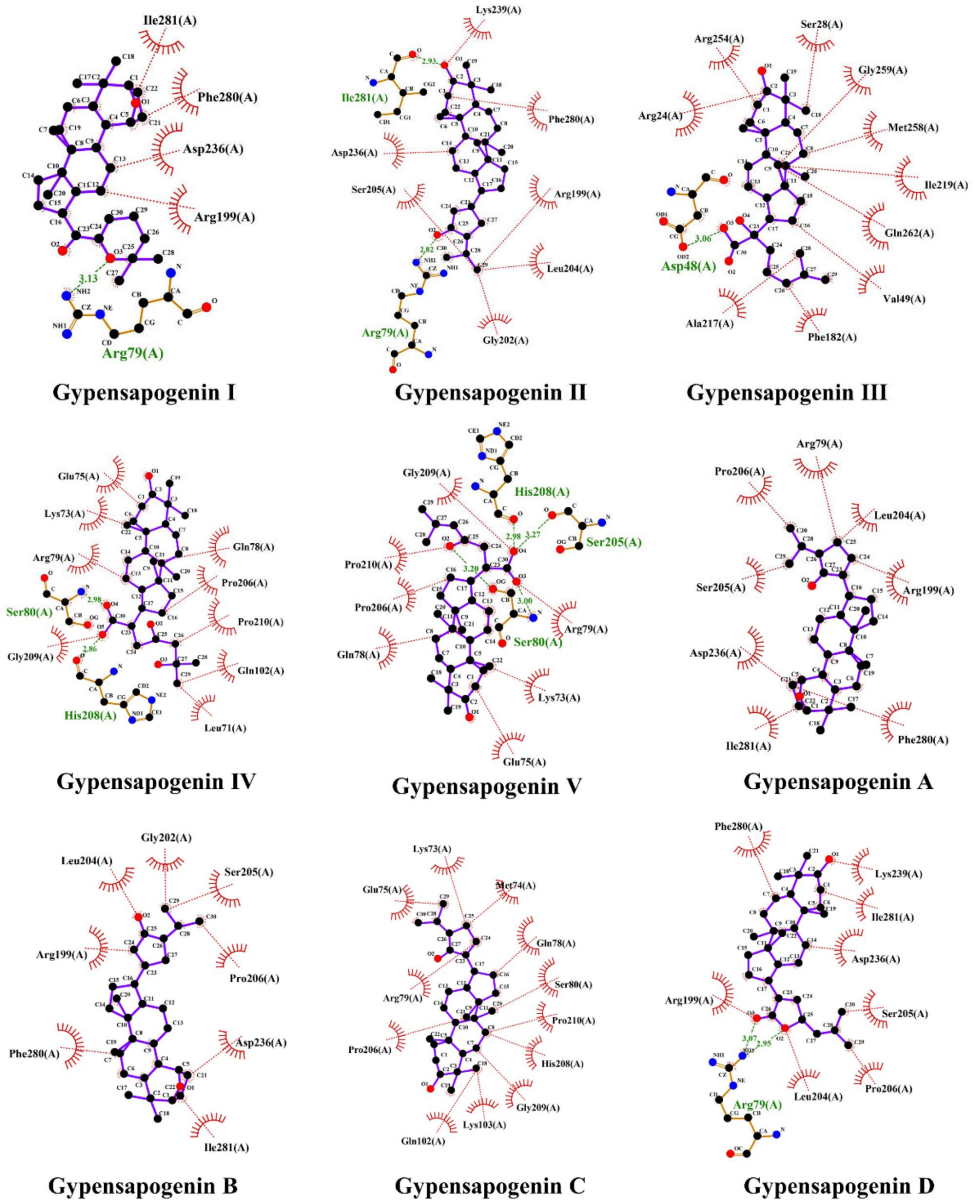
Docking pose of compounds from *G. pentaphyllum* with PTP1B target.

To further predict the PTP1B inhibitory activity of optimal dammarane triterpenoid, molecular docking was used to simulate the binding modes between PTP1B protein and the 9 dammarane-type triterpenoids from *G. pentaphyllum*, respectively. After docking, the conformational cluster with the lowest binding free energy is shown in the complex of the docked compounds with PTP1B protein (Figure 5, Table 2). The molecular docking results showed that all the triterpenoids had good affinity with the PTP1B protein (affinity ≤ −7.1 kcal/mol, Table 2). Moreover, the ligand-protein residue interactions were analysed, where hydrogen attached to the hydroxyl or the oxygen on the heterocyclic ring in all ligands except compounds Gypensapogenin A-C, showed hydrogen-bonding interaction with Arg79, Asp48, Ile281, Ser80, His208, or Ser205 active sites of residues. As shown in Table 2, Gypensapogenin III was found to have the strongest PTP1B inhibitory activity (2.34 ± 0.24 μM). In Figure 5, hydrogen-binding between the hydroxyl group on the side chain of Gypensapogenin III and Asp48 protein residues were observed with a bond distance of 3.06 Å, in addition to several hydrophobic interactions between Gypensapogenin III and other protein residues. PTP1B protein contains 435 amino acid residues, of which residues 30–278 are its catalytic domains. The active site, Asp48, can form hydrogen bonds with the phosphorylated tyrosine backbone during the catalytic process and play an important role in substrate binding and recognition^32^^,^^33^. Hence, the competitive hydrogen bonding of Gypensapogenin III with Asp48 may be a key mechanism for its PTP1B inhibitory activity.

**Table 2.** Inhibitory activity of dammarane-type triterpenoids from *G. pentaphyllum* against PTP1B.

No.	Compound name	IC_50_^a^ (μM)
1	Gypensapogenin I	20.29 ± 2.49
2	Gypensapogenin II	10.50 ± 1.07
3	Gypensapogenin III	2.34 ± 0.24
4	Gypensapogenin IV	4.26 ± 0.42
5	Gypensapogenin V	33.90 ± 0.83
6	Gypensapogenin A	19.55 ± 1.48
7	Gypensapogenin B	29.43 ± 2.48
8	Gypensapogenin C	24.60 ± 2.28
9	Gypensapogenin D	7.77 ± 0.85
10	NaVO_4_^b^	12.65 ± 1.60

^a^ IC_50_ values were expressed as means ± SD of three replications.

^b^ Positive control.

**Table 3.** The molecular docking results of dammarane-type triterpenoids from *G. pentaphyllum* against PTP1B.

No.	Compound name	Binding energy (kcal/mol)	Amino acid involved	H-bond length (Å)
1	Gypensapogenin I	−7.6	Arg79	3.13
2	Gypensapogenin II	−7.5	Arg79 Ile281	2.82 2.93
3	Gypensapogenin III	−7.1	Asp48	3.06
4	Gypensapogenin IV	−7.1	Ser80 His208	2.98 2.86
5	Gypensapogenin V	−7.0	Ser80 Ser205 His208	3.00 3.27 2.98
6	Gypensapogenin A	−7.8		
7	Gypensapogenin B	−7.5		
8	Gypensapogenin C	−7.1		
9	Gypensapogenin D	−7.8	Arg79	3.07 2.95

### ADME analysis

Drug-likeness was analysed to check whether the 9 dammarane-type triterpenoids from *G. pentaphyllum* possess favourable ADME (absorption, distribution, metabolism and excretion) properties. Drug-likeness compounds should have a good aqueous solubility, which can be predicted by three methods, including ESOL, (ALI) logS and (SILICOS-IT) logS^34^. Orally drugs should obey Lipinski’s five rules: molecular weight (MW) not more than 500 g/mol, not more than 10 hydrogen bond acceptors, not more than 5 hydrogen bond donors, LogP value less than 5, and not less than 10 rotatable bonds^35^, and compounds violating two or more of these rules are not orally active. The water solubility and intestinal membrane permeability of pharmaceuticals are their inherent qualities that affect oral absorption, according to the Biopharmaceutics Classifcation System (BCS) of the US Food and Drug Administration. Due to its function in drug dissolving within the gastric lumina, solubility is one of the important parameters that determine the oral bioavailability of drug molecules^36^. Drug-likeness analysis of 9 dammarane-type triterpenoids from *G. pentaphyllum* was listed in Table 4. All the compounds were specific in nature (zero alerts of PAINS). Among them, Gypensapogenin III showed good binding affinity against PTP1B with high drug-likeness parameters such as moderate solubility, and no excretion problems as there is no pharmacokinetic P-gp (permeable glycoprotein) interference and non-inhibitor of CYP enzymes.

**Table 4.** ADME profile of dammarane-type triterpenoids from *G. pentaphyllum* as predicted by SwissADME.

Compounds	Physicochemical properties					Solubility			Pharmacokinetics		Druglikeness	
	MW	HB donors	HB acceptors	Rotatable bonds	Consensus log P	Log S (ESOL)	Log S (Ali)	Log S (SILICOS-IT)	GI absorption	CYP enzymes inhibitors	Lipinski	Pains alert
Gypensapogenin I	452	0	3	2	5.90	Poorly soluble	Poorly soluble	Poorly soluble	Low	No	Yes	0
Gypensapogenin II	438	1	2	1	6.44	Poorly soluble	Poorly soluble	Poorly soluble	Low	No	Yes	0
Gypensapogenin III	474	3	4	5	5.79	Poorly soluble	Poorly soluble	Moderately soluble	Low	No	Yes	0
Gypensapogenin IV	490	3	5	6	5.01	Poorly soluble	Poorly soluble	Moderately soluble	High	Yes/ CYP3A4	Yes	0
Gypensapogenin V	472	2	4	5	5.78	Poorly soluble	Poorly soluble	Moderately soluble	Low	No	Yes	0
Gypensapogenin A	434	0	2	1	6.32	Poorly soluble	Poorly soluble	Poorly soluble	Low	Yes/CYP2C9	Yes	0
Gypensapogenin B	434	0	2	1	6.21	Poorly soluble	Poorly soluble	Poorly soluble	Low	Yes/CYP2C9	Yes	0
Gypensapogenin C	438	1	2	1	6.54	Poorly soluble	Poorly soluble	Poorly soluble	Low	Yes/CYP2C9	Yes	0
Gypensapogenin D	454	1	3	2	6.33	Poorly soluble	Poorly soluble	Moderately soluble	Low	Yes/CYP2C9	Yes	0

## Conclusion

In the present study, five new and four reported dammarane-type triterpenoids were isolated and identified from the hydrolysed products of total *G. pentaphyllum* saponins. Among them, compound **1** (Gypensapogenin I) is a new skeleton compound characterised as C-21 was inserted into side chain. Similar to previous reports in the literature^24^^,^^25^, all of these dammarane triterpenoid compounds showed good PTP1B inhibitory activity and the strength of PTP1B inhibitory activity was mainly related to the electron-donating group on its side chain. ADME analysis results suggested that Gypensapogenin III have a good binding affinity against PTP1B with high drug-likeness parameters. Its mechanism might be due to the formation of competitive hydrogen bonding between the electron-donating group and the Asp48 amino acid residues on the PTP1B protein.
